# Epidemiology of paediatric injuries in Rwanda using a prospective trauma registry

**DOI:** 10.1002/bjs5.50222

**Published:** 2019-11-17

**Authors:** R. T. Petroze, A. N. Martin, E. Ntaganda, P. Kyamanywa, E. St‐Louis, S. K. Rasmussen, J. F. Calland, J. C. Byiringiro

**Affiliations:** ^1^ Montreal Children's Hospital, Division of Paediatric General and Thoracic Surgery Montreal Quebec Canada; ^2^ University of Florida, Division of Pediatric Surgery Gainesville Florida USA; ^3^ Department of Surgery University of Virginia Charlottesville Virginia USA; ^4^ University of Rwanda Kigali Rwanda; ^5^ Kampala International University Kampala Uganda

## Abstract

**Background:**

Child survival initiatives historically prioritized efforts to reduce child morbidity and mortality from infectious diseases and maternal conditions. Little attention has been devoted to paediatric injuries in resource‐limited settings. This study aimed to evaluate the demographics and outcomes of paediatric injury in a sub‐Saharan African country in an effort to improve prevention and treatment.

**Methods:**

A prospective trauma registry was established at the two university teaching campuses of the University of Rwanda to record systematically patient demographics, prehospital care, initial physiology and patient outcomes from May 2011 to July 2015. Univariable analysis was performed for demographic characteristics, injury mechanisms, geographical location and outcomes. Multivariable analysis was performed for mortality estimates.

**Results:**

Of 11 036 patients in the registry, 3010 (27·3 per cent) were under 18 years of age. Paediatric patients were predominantly boys (69·9 per cent) and the median age was 8 years. The mortality rate was 4·8 per cent. Falls were the most common injury (45·3 per cent), followed by road traffic accidents (30·9 per cent), burns (10·7 per cent) and blunt force/assault (7·5 per cent). Patients treated in the capital city, Kigali, had a higher incidence of head injury (7·6 per cent *versus* 2·0 per cent in a rural town, *P* < 0·001; odds ratio (OR) 4·08, 95 per cent c.i. 2·61 to 6·38) and a higher overall injury‐related mortality rate (adjusted OR 3·00, 1·50 to 6·01; *P* = 0·019). Pedestrians had higher overall injury‐related mortality compared with other road users (adjusted OR 3·26, 1·37 to 7·73; *P* = 0·007).

**Conclusion:**

Paediatric injury is a significant contributor to morbidity and mortality. Delineating trauma demographics is important when planning resource utilization and capacity‐building efforts to address paediatric injury in low‐resource settings and identify vulnerable populations.

## Graphical abstract

This study evaluated the demographics and outcomes of paediatric injury in Rwanda through a prospective trauma registry to inform capacity‐building for prevention and treatment. Patients treated in the capital city had a higher incidence of head injury and a higher overall injury‐related mortality than those in a rural town. Pedestrians had higher overall injury‐related mortality compared with other road‐users.

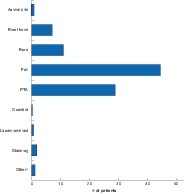

Falls and road traffic accidents significant contributors to pediatric injury in Rwanda

## Abstract (es)

**Antecedentes:**

Históricamente, las iniciativas relativas a la supervivencia pediátrica han priorizado los esfuerzos para reducir la morbilidad y la mortalidad debida a enfermedades infecciosas y patología materna. Se ha prestado escasa atención a los traumatismos en pediatría en entornos de recursos limitados. El objetivo de este estudio ha sido evaluar la demografía y los resultados de los traumatismos pediátricos en un país del África subsahariana en un intento para mejorar la prevención y el tratamiento.

**Métodos:**

Se estableció un registro prospectivo de traumatismos en dos campus universitarios de Ruanda para recoger sistemáticamente las características demográficas, atención pre‐hospitalaria, fisiología inicial y resultados, de mayo de 2011 a julio de 2015. Se efectuó un análisis univariado para los datos demográficos, mecanismos del traumatismo, localización geográfica y resultados. Para las estimaciones de mortalidad se llevó a cabo un análisis multivariable.

**Resultados:**

De un total de 11.036 pacientes incluidos en el registro, 3.010 (27,3%) tenían menos de 18 años. Los pacientes pediátricos eran predominantemente varones (69,9%) con una edad media de 8,3 años. Las caídas fueron la causa más frecuente del traumatismo (45,3%) seguidas de los accidentes de tráfico (30,9%), quemaduras (10,7%) y traumatismo cerrado/asalto (7,5%). Los pacientes tratados en la capital presentaban una incidencia más elevada de traumatismos craneales (7,5% *versus* 2,0%, *P* < 0,0001, razón de oportunidades, *odds ratio*, OR 4,08, i.c. del 95% 2,6‐6,4) y una mayor mortalidad global relacionada con el traumatismo (*P* = 0,019, OR ajustado 3,00, i.c. del 95% 1,5‐6,0). Los peatones presentaron una mortalidad global relacionada con el traumatismo más alta en comparación con otros usuarios de la carretera (*P* = 0,0074, OR ajustado 3,26, i.c. del 95% 1,37‐7,73).

**Conclusión:**

Los traumatismos pediátricos contribuyen significativamente a la morbilidad y mortalidad. Delinear la demografía de los traumatismos es importante a la hora de planificar el uso de recursos y el desarrollo de capacidades dirigidas al esfuerzo para abordar los traumatismos pediátricos en entornos de bajos recursos e identificar poblaciones vulnerables.

## Introduction

Global health initiatives have historically prioritized infectious diseases and maternal conditions in efforts to reduce child morbidity and mortality. Overall trends in global childhood mortality have decreased in the past 20 years, yet profound inequity persists among low‐ and middle‐income countries (LMICs)^1^. The Global Burden of Disease Study 2013^2^ revealed that less than 20 per cent of developing countries had achieved Millennium Development Goal 4, which aimed to reduce the under‐five mortality rate by two‐thirds between 1990 and 2015.

Surgery, including injury care, is increasingly recognized as an important component of national health plans and universal primary care^3^, ^4^, ^5^, yet over 2 billion people in low‐income countries lack access to basic, life‐saving surgical care^6^. In addition, injury‐associated mortality surpasses that of human immunodeficiency virus/acquired immune deficiency syndrome (HIV/AIDS), malaria and tuberculosis combined, accounting for 10 per cent of the world's deaths and 16 per cent of the global burden of disease^7^, ^8^, ^9^. Surgical services in general are in short supply, but surgical services for children are even more sparse in LMICs. For example, 95 per cent of injury‐related deaths in children occur in LMICs^9^.

Rwanda is a low‐income country in sub‐Saharan Africa that has made tremendous progress in improving child development indicators, with marked reductions in paediatric mortality from HIV/AIDS, tuberculosis and malaria^10^. Since 2000, the observed improvements in maternal and under‐five mortality in Rwanda have far surpassed the regional average, and approach the global mean^10^, ^11^. However, little effort has yet been devoted to paediatric surgical and trauma needs. Although the study by Wang and colleagues^2^ revealed that the annualized rate of decreasing childhood mortality in Rwanda accelerated between 1990 and the present, the burden of accidental and traumatic death on children in Rwanda remained high and did not differ significantly from estimates reported as 20 deaths per 100 000 people by the Global Burden of Disease Study 2010^12^. A Surgeons OverSeas Assessment of Surgical Need (SOSAS) countrywide household population survey study in Rwanda^13^ demonstrated that 12 per cent of 0–18‐year‐olds had potential surgical needs, of which 54 per cent were unmet.

In an effort to document and improve emergency and surgical services, a prospective trauma registry was established in Rwanda in 2011 through multi‐institutional collaboration^14^, ^15^. This study aimed to evaluate the specific demographics and outcomes of paediatric injury in Rwanda in an effort to inform prevention and treatment. The long‐term goal is to facilitate trauma prevention and treatment in children through development of a collaborative quality improvement tool, and to support the development of surgical services for children.

## Methods

Rwanda has a population of 12·2 million (4·9 million people under the age of 15 years)^16^. There are 42 district and four referral hospitals; the referral hospitals also serve as teaching hospitals^17^. Rwanda has one of the few publicly‐funded national ambulance services in sub‐Saharan Africa. Prehospital emergency medical services, known as the Service d'Aide Médicale Urgente (SAMU), were established by the Ministry of Health in 2007^18^. As few emergency or surgical services are provided at district hospitals, injured patients are often sent to the referral hospitals for evaluation and definitive care. The university teaching hospital in Kigali (Centre Hospitalier Universitaire de Kigali (CHUK)) is a public 520‐bed hospital in the capital of Rwanda. The university teaching hospital in Butare (Centre Hospitalier Universitaire de Butare (CHUB)) is a public 500‐bed hospital located 133·6 km south in Butare, Huye District, a university town with a population of 89 600 people at the 2012 census^19^.

Ethical approval for the study was obtained from the University of Virginia institutional review board for health sciences research (number 15075) and the ethics committee at CHUK (EC/CHUK/006/10).

### Registry development

Development of the Rwanda Injury Registry has been described previously^14^, ^15^, ^20^. This is a prospective trauma registry that was established at the two campuses of CHUK and CHUB in Rwanda to record systematically patient demographics, prehospital care, initial physiology and patient outcomes. Data were collected on a 31‐item paper registry form that was adapted locally for the Rwandan setting from registries in other sub‐Saharan African countries^21^, ^22^. Local training was conducted over a 1‐month period, and the form was rolled out for data collection in March 2011. Arrival data were collected by trained nurses in the accident and emergency department (ED) within 24 h of admission. Over the period of data collection, 2‐week and 1‐month in‐hospital outcome data were abstracted from patient chart review, ward registries and operating room logs by trained medical students, the principal investigator or the nurse coordinator.

Inclusion criteria included: any injured patient referred from a district hospital for injury evaluation, any injury‐related mortality in the emergency department or any inpatient hospitalization or emergency stay greater than 24 h. Patients who arrived at the first hospital of contact and were treated and sent home within 24 h were excluded.

### Statistical analysis

Data were entered into a searchable Microsoft Access® 2010 (Microsoft, Santa Rosa, California, USA) database by a trained data registrar. Deidentified data for patients aged less than 18 years were abstracted over a 4‐year period from May 2011 to July 2015. Data collected during the implementation and transition period in March and April 2011 were excluded. Descriptive analysis was performed using SAS® 9.4 (SAS Institute, Cary, North Carolina, USA). Primary outcomes assessed using univariable analysis included overall and injury‐related mortality. Outcomes were compared between hospitals and based on injury mechanism using χ^2^ and Fisher's exact tests for non‐parametric data. Multivariable logistic regression analysis was performed to control for age, sex and injury severity. Variables were selected *a priori* based on clinical relevance and available data in the registry. For example, anatomical injury severity scores were not calculated, and respiratory rate was available for only two‐thirds of the paediatric patients. Injury severity was therefore determined using the modified Kampala Trauma Score (mKTS)^23^.

## Results

**Figure 1:**
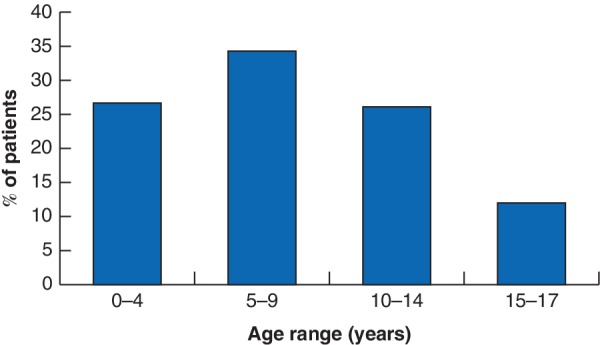
Age demographics of injured paediatric patients WHO age categories are used to describe distribution of 3010 paediatric patients in the Rwanda Injury Registry, 2011–2015.

**Figure 2:**
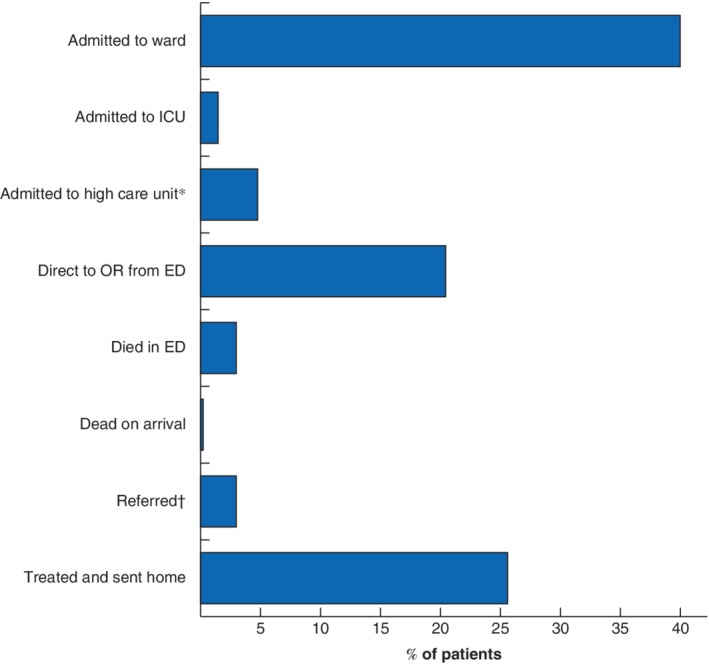
Disposition of 2928 injured paediatric patients The disposition was not available for 82 patients. *The high care unit had the availability of oxygen. †Referrals were to a higher level of care (the private hospital in the country or the military hospital). OR, operating room; ED, emergency department.

The trauma registry at the university teaching hospitals enrolled 11 036 patients between May 2011 and July 2015. More than one‐quarter of these patients (3010, 27·3 per cent) were under the age of 18 years. The overall paediatric injury‐related mortality rate was 4·8 per cent (144 patients). Patients were predominantly male, and the median age was 8 (i.q.r. 5–12) years. The majority of patients were aged 5–9 years (1042, 34·6 per cent) (*Fig*. 1). Additional demographic and clinical factors used to calculate the mKTS can be found in *Table* 1. Most patients presenting with a trauma mechanism were admitted to the hospital (1974 of 2928, 67·4 per cent), and just over one‐quarter (751, 25·6 per cent) received treatment in the ED and were sent home (*Fig*. 2).

**Table 1:** Demographic characteristics

	No. of patients (*n* = 3010)*
**Age (years)** †	8 (5–12)
< 5	810 (26·9)
5–17	2200 (73·1)
**Sex**	(*n* = 2996)
F	902 (30·1)
M	2094 (69·9)
**Hospital**	
Butare	1171 (38·9)
Kigali	1839 (61·1)
**mKTS** †, ‡	12 (11–12)
**No. of serious injuries**	(*n* = 2977)
0	242 (8·1)
1	2556 (85·9)
> 1	179 (6·0)
**Systolic blood pressure (mmHg)**	(*n* = 2453)
> 89	2361 (96·2)
50–89	91 (3·7)
< 50	1 (0·04)
Undetectable	0 (0)
**Neurological status**	(*n* = 2950)
Alert	2324 (78·8)
Responds to verbal stimuli	372 (12·6)
Responds to painful stimuli	209 (7·1)
Unresponsive	45 (1·5)

* With percentages in parentheses unless indicated otherwise;

† values are median (i.q.r.).

‡ The modified Kampala Trauma Score (mKTS) was measured in 2098 patients; elements include age range, number of serious injuries, BP range and neurological status, for a total score of 5–16.

**Figure 3:**
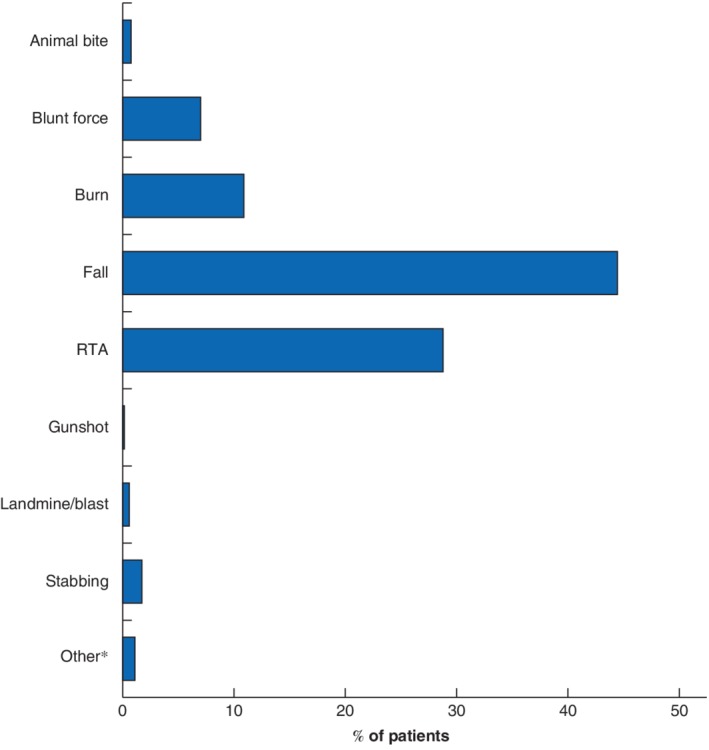
Causes of injury in 2982 paediatric patients in Rwanda, May 2011 to July 2015 The mechanism of injury was missing for 28 children. *Includes choking (1 patient), near‐drowning (3), electrical injury (2), sexual assault (2) and non‐specified (27). RTA, road traffic accident.

Falls were the most common cause of injury (1351 of 2982, 45·3 per cent), followed by road traffic accidents (RTAs) (921 of 2982, 30·9 per cent), burns (348, 10·7 per cent) and blunt force/assault (224, 7·5 per cent) (*Fig*. 3). Of the 1974 admitted patients, 599 (30·3 per cent) went directly to the operating room from the ED.

In univariable analysis there was a statistically significant difference in overall mortality for patients who sustained injury as a result of an RTA (55 of 921, 6·0 per cent) compared with other causes of injury (OR 1·43, 95 per cent c.i. 1·01 to 2·02; *P* = 0·043). Pedestrians are a particularly vulnerable category, with 58·1 per cent of children (535 of 921) involved in an RTA being pedestrians. Pedestrians involved in an RTA were more likely to die than non‐pedestrians (7·9 per cent (42 of 535) *versus* 3·4 per cent (13 of 386) respectively, *P* = 0·005; OR 2·44, 1·29 to 4·62). In addition, for all children involved in an RTA, the rate of severe head injury trended towards significance in pedestrians (12·3 per cent (66 of 535) *versus* 8·5 per cent (33 of 386) in non‐pedestrians, *P* = 0·067; OR 1·51, 0·97 to 2·34). Falls were associated with a lower overall injury‐related mortality (1·7 per cent (23 of 1351) *versus* 7·3 per cent (121 of 1659) for other causes of injury, *P* < 0·001; OR 0·22, 0·14 to 0·35).

In multivariate analysis, controlling for mKTS, age and sex, mortality from RTAs (compared with other causes) was not significantly different. However, pedestrians involved in an RTA had an increased risk of death compared with other road‐users (adjusted OR 3·26, 95 per cent c.i. 1·37 to 7·73; *P* = 0·007). Falls again had a lower overall injury‐related mortality rate when controlling for age, sex and injury severity (adjusted OR 0·43, 0·24 to 0·76; *P* = 0·004).

There were significant differences between outcomes for patients treated at CHUK, based in the capital city of Kigali, *versus* CHUB, located in a rural town. Patients treated at CHUK had a higher incidence of head injury (7·6 per cent *versus* 2·0 per cent for CHUB, *P* < 0·001; OR 4·08, 95 per cent c.i. 2·60 to 6·40) and a higher overall injury‐related mortality rate (6·9 per cent (127 of 1830) *versus* 1·5 per cent (17 of 1171) respectively, *P* < 0·001; unadjusted OR 5·00, 3·00 to 8·40; adjusted OR 3·00, 1·50 to 6·01). Additionally, at CHUK, burn injuries (15·0 per cent (273 of 1823) *versus* 6·5 per cent (75 of 1159) respectively), blunt force trauma (8·0 (146 of 1823) *versus* 6·7 per cent (78 of 1159)) and RTAs (36·8 per cent (671 of 1823) *versus* 21·6 per cent (250 of 1159)) were more common compared to other causes (OR 2·10, 1·79 to 2·51; *P* < 0·001). Conversely, fall injuries were more common at CHUB (60·9 per cent (706 of 1159) *versus* 35·4 per cent (645 of 1823) for CHUK, *P* < 0·001; OR 0·36, 0·31 to 0·41). The mortality rate in the ED also differed significantly between the two hospitals: 95 of 1839 (5·2 per cent) at CHUK compared with only three of 1171 (0·3 per cent) at CHUB (*P* < 0·001) (unadjusted OR 21·2, 6·70 to 67·10). Other differences in injury epidemiology and outcomes between the two hospitals are shown in *Table* 2.

**Table 2:** Comparison of cause of trauma and outcome by hospital in 3010 patients

	Butare (*n* = 1171)	Kigali (*n* = 1839)	*P* ¶	Unadjusted OR*	Adjusted OR*, §	Adjusted *P*
Severe head injury†	23 (2·0)	139 (7·6)	< 0·001	4·08 (2·61, 6·38)	1·24 (0·65, 2·38)	0·518
Mortality‡	17 (1·5)	127 (6·9)	< 0·001	5·04 (3·02, 8·40)	3·00 (1·50, 6·01)	0·019
Fall	706 (60·3)	645 (35·1)	< 0·001	0·36 (0·31, 0·41)	0·43 (0·36, 0·51)	< 0·001
Road traffic injury	250 (21·3)	671 (36·5)	< 0·001	2·12 (1·79, 2·51)	1·99 (1·62, 2·44)	< 0·001
Severe head injury from road traffic injury	18 (7·2)	86 (12·8)	< 0·001	2·68 (1·47, 4·90)	0·87 (0·38, 2·03)	0·753
Mortality from road traffic injury	4 (1·6)	51 (7·6)	< 0·001	5·06 (1·81, 14·15)	4·37 (1·01, 18·92)	0·048

Values in parentheses are percentages unless indicated otherwise;

* values in parentheses are 95 per cent confidence intervals.

† Defined as Glasgow Coma Scale score at admission of 3–8;

‡ defined as all‐cause 30‐day in‐hospital mortality.

§ Controlling for age, sex and modified Kampala Trauma Score.

OR, odds ratio.

¶ χ^2^ or Fisher's exact test.

## Discussion

Regionally developed hospital‐based trauma registries have been shown to be effective mechanisms for injury surveillance in resource‐limited settings^21^, ^24^, ^25^, ^26^. They also allow the development of locally relevant and properly adapted metrics for patient‐level risk adjustment and severity scoring, such as the Kampala Trauma Score (KTS)^27^, ^28^. This study has demonstrated that the Rwanda Injury Registry was successful in documenting the injury epidemiology and outcome of children with injuries presenting at the two main referral hospitals in the country. Previous studies from the USA and the UK demonstrated that injuries secondary to RTAs and falls are common causes of trauma in children^29^, ^30^. However, the present study revealed higher mortality, head injury and proportion of injuries due to RTAs in the capital, Kigali. This may be secondary to a larger and growing urban population as well as increased traffic and congestion in the capital. However, Rwanda also has an active emergency medical response service^31^, so it may be possible that increased presentation of severe head injury in the capital is due to the availability of emergency services in the city. This offers the opportunity in the future for coordination of prehospital evaluation with in‐hospital outcomes, as well as evaluation over time.

The mortality variables in the present data, controlling for injury severity, are presented with caution. Several studies^27^, ^28^, ^32^ have documented the appropriateness of using the KTS, an aggregate of systolic BP, neurological status, age and respiratory rate, to stratify injury severity. The mKTS eliminates respiratory rate, owing to the unreliability of its recording in many LMICs^28^, ^33^, and was used to risk‐stratify injured adult patients in a previous study^34^ that used Rwanda trauma registry data. Using physiological variables as categorized in the KTS to control for injury severity may present certain limitations for the population being studied in this analysis. Missing data and inaccuracies were sought by periodic data audits; however, these reviews were intermittent and relied on traditional chart review for rectification, a process that is itself not immune to error. Only two‐thirds of the patients were included in the adjusted estimates owing to missing variables. It was hypothesized that severely injured or moribund patients who died in the ED may have missing values. In examining data missingness, however, it was found that, contrary to expectations, the proportion of incomplete variables was higher in walking wounded patients than in severely injured patients. In either scenario, an uncontrolled selection bias may have been created. Furthermore, the KTS has never been validated in a paediatric trauma population, and does not account for the different physiology and haemodynamic status of young children. For this reason, age was not included as a continuous variable in the present multivariable analysis.

Further, the present study revealed increased transfer of head‐injured patients from Butare. This is likely due to the fact that, at the time of data collection, neurosurgical and CT services were available only in the capital. CHUB has since obtained a CT scanner, but there remain challenges in operation. As emergency and surgical capacity develops in the country, continued assessments will be imperative for sustained improvements in the quality of care delivery. Efforts to reinforce the research and data collection infrastructure in Rwanda, such as ongoing trauma registry revitalization and implementation of other registries tracking the incidence and outcomes of surgical care for cancer and congenital anomalies, are crucial to sustained growth and improvements in quality. Since this study was conducted, a paediatric surgery unit has been established with a full‐time paediatric surgeon and a dedicated operating room for paediatric cases. The emergency medicine residency programme has graduated its first residents, and there are ongoing efforts to improve the multidisciplinary care of injured patients, including incorporation of injury tracking at the national health plan level.

Limitations of this study include variable quality of chart documentation with missing or incomplete documentation, inconsistent languages used, or loss of information. To mitigate these, an assigned member of the research team monitored the data collection process through separate chart review. However, the lack of personnel with time to prioritize documentation or data collection is a major issue for sustainable data collection, and this significantly affected the continuity of the present study. In addition, the design of the current registry can assess outcomes only in hospital and for up to 1 month after initial presentation, and cannot determine long‐term outcomes and disability. Furthermore, by limiting the study to the two university referral hospitals, the true burden of injury and disability at the community level could not be captured to inform targeted prevention fully, particularly as other hospitals increase their capacity to accept referrals from rural settings.

This study has shown that paediatric injury is a significant contributor to morbidity and mortality in Rwanda. This unfortunate trend in LMICs, forecasted by the Global Burden of Disease and risk factors study, has also been demonstrated by more recent reports^35^, ^36^. The present study identified a significant vulnerable population – paediatric pedestrians – that could benefit from targeted intervention and prevention efforts. This has been correlated in separate prehospital analyses in Rwanda^37^. Identification of target populations that suffer disparate outcomes, such as paediatric mortality in pedestrians, is important for ongoing public health sector development.
